# Influence of dietary lysolecithin on growth performance, nutrient digestibility, haemato-biochemistry, and oxidative status of broiler birds

**DOI:** 10.1007/s11250-024-04107-7

**Published:** 2024-09-23

**Authors:** Augustine Ogbonna Ani, Matthew Onyeka Onodugo, Mercy Chisara Ogwuegbu, Valentine Chidozie Udeh, Doctor Mziwenkosi Nhlanhla Mthiyane

**Affiliations:** 1 Department of Animal Science, Faculty of Agriculture, University of Nigeria, Nsukka, Enugu State 410001 Nigeria; 2 Food Security and Safety Focus Area, Faculty of Natural and Agricultural Sciences, North-West University, Mafikeng Campus), Private Bag X2046, Mmabatho, South Africa; 3 Department of Agricultural, Food and Nutritional Science, University of Alberta, Edmonton, AB T6G 2P5 Canada; 4 Department of Animal Science, Faculty of Natural and Agricultural Sciences, School of Agricultural Sciences, North-West University (Mafikeng Campus), Private Bag X2046, Mmabatho, South Africa

**Keywords:** Lysolecithin, Broiler, Performance, Nutrient digestibility, Antioxidant

## Abstract

This study examined the effect of dietary lysolecithin on growth performance, nutrient digestibility, haematobiochemistry, and oxidative status in finisher broiler chickens. In a completely randomized design (CRD), 300 21-day-old *Chikun strain* chicks were randomly allocated to 30 pens in which they were allotted to 5 dietary treatments as follows TI (0 g /100kg), T2 (100 g /100kg), T3 (200 g /100kg), T4 (300 g /100kg), T5 (400 g /100kg) of feed with 6 replicates pens of 10 during the finisher phase (28 days). Results showed that dietary supplementation with lysolecithin increased the final body weight (FBW) (quadratic *P =* 0.0178), body weight gain (BWG) (quadratic *P =* 0.0232), whilst it decreased the total feed intake (TFI) (linear *P* = 0.0104). Similarly, it linearly increased the retention of dry matter (*P =* 0.0324); crude protein (*P =* 0.0029), crude fibre (*P =* 0.0147), and crude fat (*P =* 0.0002). Furthermore, it increased the superoxide dismutase (linear *P* < 0.001), glutathione peroxidase (quadratic *P* < 0.001), glutathione (linear *P* < 0.001), whilst it decreased malondialdehyde (linear *P* = 0.003), without affecting (*P* > 0.05) the haematobiochemistry parameters. Therefore, dietary lysolecithin could be supplemented up to 400 g /100 kg without compromising performance, nutrient retention, haemato-biochemistry, and oxidative status in finisher broiler diets.

**Supplementary Information:**

The online version contains supplementary material available at 10.1007/s11250-024-04107-7.

## Introduction

In broiler chicken production, diet plays a vital role in their growth and development. The nutrient density of the diet is a crucial determinant that has a substantial impact on the growth and overall health of broilers. This aspect directly influences the economic viability of broiler production (Kairalla et al. 2023; Gholami et al. 2024). As a result, researchers have consistently sought innovative dietary strategies that can efficiently reduce the high cost of feed through metabolizable energy intake reduction while simultaneously ensuring optimal performance and health of broiler chickens (Alshelmani et al. 2016; Gholami et al. 2024).

Enhancing fat digestibility is a viable means of reducing supplementary lipids in broiler diets, resulting in low feed production costs without adversely affecting the performance of broiler chickens (Majdolhosseini et al. 2019; Ahmadi-Sefat et al. 2022). In this context, incorporating emulsifiers such as lysolecithin into broiler diets represents a targeted approach to achieving this goal (Jaapar et al. 2020). It could potentially address the challenges related to lipid digestion within the digestive tract (Zampiga et al. 2016; Boontiam et al. 2017; Mohammadigheisar et al. 2018).

Lysolecithin is derived from lecithin, which is a type of fatty substance found naturally in several food sources, such as whole grains, egg yolk, soybean, and milk (Liu et al. 2020). It plays a crucial role in enhancing the efficiency of fat digestion by increasing the surface area, which aids the activity of lipase enzymes. These enzymes work to hydrolyze triglycerides molecules into monoglycerides and fatty acids, facilitating the formation of micelles containing products of lipid breakdown. This mechanism is crucial in lipid absorption because it creates a diffusion gradient that promotes efficient digestion and lipid absorption (Guerreiro Neto et al. 2011).

Several studies have demonstrated that supplementation of lysolecithin in broiler diets improves the formation of micelles and emulsions by aiding bile salts, thereby enhancing lipid digestibility and overall performance in broilers (Joshi et al. 2006; Gholami et al. 2024). Also, dietary supplementation of lysolecithin in broiler diet has been shown to improve performance, nutrient utilization, blood metabolites, and oxidative status (Zampiga et al. 2016; Boontiam et al. 2017; Haetinger et al. 2021; Ahmadi-Sefat et al. 2022). Additionally, there is scarcity of data regarding increasing levels of lysolecithin on oxidative parameters. Thus, this study aimed to address these gaps by examining the effects of dietary lysolecithin supplementation on growth performance, nutrient digestibility, haemato-biochemistr**y**, and oxidative status in broiler chickens. Our hypothesis assumes that feeding broilers with lysolecithin-containing diets will yield improvements in these parameters, ultimately enhancing health and overall performance.

## Materials and methods

### Study location and sourcing of research materials

The study was carried out at the Poultry Unit, Department of Animal Science, University of Nigeria, Nsukka, Nigeria. Lysolecithin was purchased from Feedserve Solutions Limited, Kemin Industries, South Africa (PTY) LTD, whilst other dietary ingredients were supplied by Sabugo Feed Mill Limited Nsukka, Enugu State, Nigeria.

### Birds, design, diet, and chemical analysis

Twenty-one-day-old male *Chikun* strain chicks (729.06 g ± 3.16) were purchased from Agritted Hatchery (Ibadan, Nigeria). In a Completely Randomized Design, 300-day-old chicks were randomly allocated to 30 pens (2.6 m width x 3 m length), in which they were allotted to 5 dietary treatments, each with 6 replicate pens of 10 birds, for 28 days. Clean feed and water were offered everyday *ad libitium* throughout the feeding trial. The experimental diets were formulated to produce 5 iso-caloric and iso-nitrogenous with incremental levels of lysolecithin (0, 100, 200, 300, and 400 g /100kg), to meet the nutritional requirements of broiler finisher chickens (Table 1) (NRC 1994). Afterwards, 100 g of experimental diets were milled (2 mm sieve) and analysed using standard (AOAC 2005) methods. The dry matter was determined by oven drying 1 g of sample in pre-weighed crucibles for 12 h at 105 °C. Samples were removed from the oven and placed in a desiccator to cool, then weighed. We then calculated the dry matter as the difference between the initial sample weight and the moisture weight and expressed it as g/kg. The crude protein was determined following the standard Kjeldahl method and multiplying nitrogen values by 6.25. An ANKOM2000 FibreAnalyser with 0.255 N crude fibre solution and 0.313 N crude fibre base solution were used to analyze crude fibre. Crude fat was determined by ether extraction of crude fat using an automated Soxhlet Fat Analyser ANKOMXT15 extractor, following the operator’s manual (ANKOM Technology, Macedon, NY, USA).

**Table 1:** Ingredient and chemical composition (%, unless stated otherwise) of experimental diets for broiler finisher chickens (d21–49)

Ingredients	T1	T2	T3	T4	T5
Yellow maize	45.00	45.00	45.00	45.00	45.00
Soybean meal	30.50	30.50	30.50	30.50	30.50
Palm kernel cake	12.32	12.32	12.32	12.32	12.32
Fish meal	4.70	4.70	4.70	4.70	4.70
Bone meal	4.00	4.00	4.00	4.00	4.00
Limestone	2.50	2.50	2.50	2.50	2.50
Iodized salt	0.25	0.25	0.25	0.25	0.25
Vitamin-mineral premix^1^	0.26	0.26	0.26	0.26	0.26
Methionine	0.24	0.24	0.24	0.24	0.24
Lysine	0.23	0.23	0.23	0.23	0.23
**Total**	100.00	100.00	100.00	100.00	100.00
**lysolecithin (g)**	0.00	100	200	300	400
**Calculated composition**					
Metabolizable energy (MJ/kg)	12577.42	12577.42	12577.42	12577.42	12577.42
Crude protein	20.85	20.85	20.85	20.85	20.85
Crude fibre	5.23	5.23	5.23	5.23	5.23
Calcium	1.47	1.47	1.47	1.47	1.47
Phosphorus	0.69	0.69	0.69	0.69	0.69
**Chemical composition (%)**					
Dry matter	89.13	88.95	89.08	89.05	89.10
Crude protein	20.01	20.00	20.03	20.04	20.02
Ether extract	3.01	3.00	3.03	3.00	3.02
Crude fibre	5.04	5.10	5.05	5.05	5.05
Ash	4.11	4.07	4.10	4.05	4.10
Nitrogen free extract	67.83	67.83	67.79	67.86	67.81

TI (0 g/100kg), T2 (100 g/100kg), T3 (200 g/100kg), T4 (300 g/100kg), T5 (400 g/100kg) of feed 1Mineral−vitamin premix: Each 1.5 kg of premix contained: 0.75 g Vitamin BI, 5 g Vitamin B2, 10,000.00 IU Vitamin A, 2,000 IU Vitamin D3, 0.050 g, 2–0.015 g Vitamin B1 12.5 g Biotin, 2.5 g K3, 1 g Folic acid, 12.5 g Calcium pantothenate, 64 g Manganese, 0.8 g Iodine, 250 g Choline chloride, 0.8 g Cobalt, 25 g Vitamin E, 0.6 g Selenium, 120 g Lysine, 50 g DL−Methionine, 8 g Copper, 40 g Zinc, 32 g Iron, and 25 g Nicotinic acid

### Growth performance

Feed offered and refusals were recorded for the calculation of feed intake (FI), and the weekly body weight gain (BWG) was calculated by subtracting the current body weight from the previous weight and divided by the number of birds per pen. Next, the feed conversion ratio (FCR) was calculated by dividing the average feed intake by the average weight gain.

### Nutrient digestibility trial

A week before the end of the feeding trial, two birds were randomly selected from each pen and were placed in cleaned metabolism cages. They were adapted for 3 days before the 4-day sample (droppings) collection. Samples were dried at room temperature, ground, and analyzed for dry matter (method 930.15), crude protein (method 954.01), ether extract (method 920.39), and crude fibre (AOAC 2005). The nutrient digestibility of each of the measured nutrients was calculated using the formula:

$$\begin{aligned}Nutrient\:digestibility\:\left(\%\right)&=\frac{Nutrient\:in\:feed\:-Nutrient\:in\:faeces\:\:\:\:\:}{Nutrient\:in\:feed}\\& \quad\:\times\:100\end{aligned}$$

### Haemato-biochemistry

On day 49, a 21-gauge needle was used to collect blood from the wing veins of 2 birds per pen. The blood was placed in purple-top EDTA-coated vacutainer tubes and analysed for haematology (haemoglobin, white blood cells, red blood cells, platelets, packed cell volume, neutrophils, lymphocytes, monocytes, eosinophils, and basophils) using an automated IDEXX LaserCyteHematology Analyser, whilst blood collected into a red top Vacuette^®^ Serum Clot Activator tubes without EDTA (Greiner BioOne, GmbH, Frickenhausen, Germany) was analysed for biochemistry (urea, total protein, creatinine, aspartate aminotransferase, alanine aminotransferase, alkaline phosphatase) using an automated IDEXX catalyst one analyser.

### Antioxidant activity assays

From each of the six replicates, two birds were selected randomly. Ten grams (10 g) of the breast meat that was taken from each slaughtered bird per replicate was used for antioxidant enzyme activities and lipid peroxidation such as malonaldehyde, superoxide dismutase, catalase, glutathione peroxidase, and glutathione (MDA, SOD, GPx, and GSH), respectively. As described by (Jensen et al. 1997) using a spectrophotometer to monitor the absorbance change at 532 nm with 2-TBA, the malonaldehyde (MDA) level was analyzed. With a slight modification, SOD, catalase, GPx, and GSH activities were assayed according to the method of (Jollow et al. 1974; Aebi 1984; Kakkar et al. 1984).

### Statistical analysis

Data were analysed using a one-way analysis of variance (ANOVA) in general linear model (GLM) procedure of (SAS 2002), where treatment was the main factor. For all the parameters measured, significance was considered at *P* ≤ 0.05, and the mean among the treatment groups was separated using Least-Square Means. Then, the growth performance, nutrient digestibility, haemato-biochemistry, and oxidative status were analysed for linear and quadratic effects using polynomial contrasts. The quadratic model used to predict the optimum lysolecithin levels using response surface regression analysis is:

### Y = ax2 + bx + c

where *y* = response variable, *a* and *b* = coefficients of the equation, *c* = intercept, *x* – lysolecithin levels, and –*b*/2*a* = x value for optimal response.

## Results

### Growth performance

The effect of dietary lysolecithin incorporation on the growth performance of broiler chickens is shown in Table 2. Results showed that lysolecithin increased final body weight (FBW) (quadratic: *P =* 0.0178), body weight gain (BWG) (quadratic: *P =* 0.0232), whilst total feed intake (TFI) was decreased (linear: *P* = 0.0104). However, feed conversion ratio (FCR) showed neither a linear nor a quadratic effect (*P >* 0.05).

**Table 2:** Growth performance of finisher broiler chickens fed diets incorporated with incremental levels of lysolecithin

Lysolecithin (g/100kg)	0	100	200	300	400		Significance	
Variables	T1	T2	T3	T4	T5	SEM	*P*-_Linear_	*P*-_Quadratic_
IBW (g)	706.63	745.83	738.33	718.77	735.43	19.39	0.6267	0.1991
FBW (g)	2273.25^c^	2349.87^b^	2409.92^a^	2445.98^a^	2457.22^a^	111.1	0.7512	0.0178
BWG(g)	1566.63^c^	1604.04^b^	1671.59^a^	1727.21^a^	1721.79^a^	121.99	0.8252	0.0232
TFI(g)	4373.88^a^	4407.2^a^	4373.88^a^	4290.72^b^	4340.56^b^	105.2	0.0104	0.8181
FCR	2.79	2.74	2.61	2.48	2.52	0.23	0.8579	0.5966

^a, b,c^Means on the same row with common superscripts do not differ (*P <* 0.05); IBW = Initial body weight; FBW = final body weight; BWG = body weight gain; TFI = total feed intake; FCR = feed conversion ratio; SEM = standard error of the mean

### Nutrient digestibility

Table 3 shows the effect of lysolecithin-supplemented diets on the apparent nutrient metabolizability of finisher broiler chickens. Dietary feeding of incremental levels of lysolecithin increased dry matter (linear: *P* = 0.0324), crude protein (linear: *P* = 0.0029), crude fibre (linear: *P* = 0147), and crude fat (linear: *P* = 0.0002).

**Table 3:** Nutrient digestibility (%) of finisher broiler chickens fed diets incorporated with incremental levels of lysolecithin

Lysolecithin (g/100kg)	0	100	200	300	400		Significance	
Variables	T1	T2	T3	T4	T5	SEM	*P*-_Linear_	*P*-_Quadratic_
Dry matter	71.61^b^	74.85^b^	81.66^ab^	85.34^a^	79.27^ab^	3.63	0.0324	0.1108
Crude protein	61.66^c^	68.17^b^	79.53^ab^	81.79^a^	79.56^ab^	4.55	0.0029	0.1213
Crude fibre	15.91^b^	16.09^b^	16.44^b^	18.87^a^	18.94^a^	1.07	0.0147	0.6196
Crude fat	49.81^b^	50.05^b^	54.91^ab^	59.88^a^	59.84^a^	1.81	0.0002	0.9651

^a, b, c^ Means on the same row with common superscripts do not differ (*P <* 0.05). SEM = standard error of the mean

### Haemato-biochemistry parameters

The effects of dietary incorporation of lysolecithin on the haemato-biochemistry of broiler chickens are presented in Tables 4 and 5. The results showed that dietary inclusion of lysolecithin induced no linear or quadratic effects (*P* > 0.05) on all haemato-biochemistry.

**Table 4:** Haematological parameters of finisher broiler chickens fed diets incorporated with incremental levels of lysolecithin

Lysolecithin (g/100kg)	0	100	200	300	400		Significance	
Variables	T1	T2	T3	T4	T5	SEM	*P*-_Linear_	*P*-_Quadratic_
HB (g/dL)	10.60	10.27	10.53	10.43	10.27	0.09	0.4302	0.3241
WBC (x10mm^3^)	9500.01	9166.67	9600.01	9266.66	9500.03	155.55	0.7587	0.6412
RBC (x10^6^)	9.79	10.39	10.37	10.12	10.27	0.41	0.4415	0.3491
Platelet(K/µL)	1640.01	1677.66	1650.01	1567.67	1650.02	52.12	0.3220	0.6224
PCV (%)	36.03	36.33	39.05	38.67	38.67	2.57	0.2319	0.5654
Neutrophy (%)	20.05	21.33	21.33	21.33	21.5	1.42	0.4237	0.5961
Lymphocyte (%)	80.05	83.33	80.33	78.00	76.67	3.95	0.2991	0.5202
Monocyte (%)	2.00	2.00	2.00	2.00	2.00	0.82	1.0000	1.0000
Eosinophil (%)	1.05	1.33	1.67	1.33	2.04	0.31	0.7427	0.7813
Basophil (%)	0.51	0.67	0.67	0.67	0.67	0.34	1.0000	1.0000

Means within a row with different superscripts are significantly different (*P* < 0.05); HB = haemoglobin; WBC = white blood cell; RBC = red blood cell; PCV = packed cell volume; SEM = standard error of the mean

**Table 5:** Serum biochemistry parameters of broiler chickens fed diets incorporated with incremental levels of lysolecithin

Lysolecithin(g/100kg)	0	100	200	300	400		Significance	
Variables	T1	T2	T3	T4	T5	SEM	*P*-_Linear_	*P*-_Quadratic_
Urea (mg/dL)	63.51	61.02	60.33	59.33	61.33	3.48	0.4988	0.4253
Total protein (mg/L)	6.29	6.44	6.51	6.19	6.26	0.25	0.5890	0.6002
Creatinine (mg/dL)	3.29	3.35	3.09	3.12	2.92	0.27	0.0805	0.2655
AST (IU/L)	35.21	35.67	37.31	35.67	36.52	1.08	1.0000	0.7429
ALT (IU/L)	25.56	25.33	25.61	25.67	25.33	0.91	0.6969	0.9124
ALP (IU/L)	54.00	52.67	54.00	54.18	56.67	2.21	0.6699	0.7183

^ab^ Means within a row with different superscripts are significantly different (*P* < 0.05); AST = aspartate aminotransferase; ALT = alanine aminotransferase; ALP = alkaline phosphatase; SEM = standard error of the mean

### Anti-oxidant enzyme activity

Table 6 displays the effect of dietary lysolecithin on broiler chicken anti-oxidative enzyme activity parameters. Our data showed that dietary lysolecithin decreased malondialdehyde (linear *P* = 0.003), whilst increasing superoxide dismutase (linear *P <* 0.001), glutathione peroxidase (quadratic: *P* < 0.001), and glutathione (linear: *P* < 0.001).

**Table 6:** Antioxidative status of finisher broiler chickens fed diets incorporated with incremental levels of lysolecithin

Lysolecithin (g/100kg)	0	100	200	300	400		Significance	
Variables	T1	T2	T3	T4	T5	SEM	*P*-_Linear_	*P*-_Quadratic_
MDA(mg/dl)	4.89^a^	3.76^b^	3.50^b^	3.75^b^	3.51^b^	0.13	0.0003	0.0058
SOD (IU/L)	8.33^d^	10.48^c^	13.34^b^	16.36^a^	17.62^a^	0.36	< 0.0001	0.3184
Catalase (mg/dl)	0.43	0.46	0.43	0.45	0.43	0.01	0.4601	0.7241
GPx (mg/dl)	0.52^c^	0.72^b^	0.88^ab^	0.96^a^	0.97^a^	0.01	< 0.0001	< 0.0001
GSH (mg/dl)	2.06^c^	2.36^c^	3.37^b^	5.03^a^	5.37^a^	0.38	< 0.0001	0.9810

^abcd^Means on the same row with common superscripts do not differ (*P <* 0.05). SEM = standard error of the mean. MDA = malondialdehyde; SOD = superoxide dismutase; GPx = glutathione peroxidase; GSH = glutathione

## Discussion

A closer examination through regression analysis revealed the potential to optimize body weight gain (BWG) and feed intake (FI) through the inclusion of lysolecithin in the broiler diet. The observed improved performance effect of dietary lysolecithin supplementation was evident in an increased quadratic response in BWG and a linear decrease in TFI, particularly at the higher inclusion levels of 300 and 400 g /100kg diet. The increased BWG at these higher levels indicates that the energy and protein content were effectively utilized by the animals for maintenance and production. Broilers can regulate their energy intake by adjusting their feed intake as their diet’s energy concentration changes, which explains the reduced FI. These findings are consistent with previous reports of increased BWG in broilers fed lysolecithin (Zhang et al. 2011; Zhao and Kim 2017; Zang et al. 2022). Several factors may contribute to the enhancement in growth performance. Lysolecithin’s ability to act as an emulsifier improves fat digestion and absorption, leading to better energy utilization. Dietary emulsifiers can enhance the emulsification process by stabilizing and clearing the lipid droplet surface with the help of bile salts, which allows lipase to bind to the interphase. This process not only aids in fat digestion but also stimulates appetite and improves feed utilization efficiency, possibly through increased fatty acid digestibility (Zhang et al. 2011; Siyal et al. 2017; Zhao and Kim 2017). Additionally, including an emulsifier in the diet may enhance the adsorption-desorption balance, which is influenced by amphiphilic molecules like fats, phospholipids, and proteins (Majdolhosseini et al. 2019). Consequently, the changes induced by the external emulsifier may lead to improved nutrient absorption across the enterocyte membrane, thereby increasing the bioavailability of nutrients in the feed. Moreover, dietary supplementation with lysolecithin may improve gut health and integrity, reducing digestive stress and optimizing nutrient uptake. This contributes to more efficient feed conversion and reduced feed intake. Furthermore, lysolecithin’s ability to modulate lipid metabolism and reduce bile acid deconjugation could enhance fat utilization, thereby improving the growth performance in broilers fed low density diets (Papadopoulos et al. 2018). The observed improvement in growth performance following lysolecithin supplementation aligns with the increased digestibility of crude fat and crude protein noted in this study.

Moreover, dietary lysolecithin linearly increased the metabolizability of dry matter, crude protein, crude fibre, and crude fat. These findings suggest that higher incremental levels of lysolecithin (200–400 g /100kg) are superior to lower levels (100 g /100kg) in enhancing nutrient digestibility. The increased inclusion of lysolecithin improved the digestibility coefficients of dry matter, crude protein, crude fibre and crude fat. This observed enhancement in nutrient digestibility could be attributed to lysolecithin’ role as both an energy source and an emusifer. Additionally, lysolecithin helps to slow the passage of food through the digestive system, allowing for greater nutrient absorption and digestion within the digestive tract (Ravindran et al. 2016). Previously published studies, by Jansen (2015) and Zhao and Kim (2017) reported similar outcomes, suggesting that improved nitrogen retention could result from the emulsification properties of lysolecithin. Lysolecithin enhances oil dispersion in water, with a higher hydrophilic/lipophilic balance (HLB) value compared to lecithins, which enhance water dispersion in oil. During digestion, the intestinal tract effectively emulsifies lipids with oil dispersed in water, thereby facilitating fat digestion and absorption (Omer and Chiodi 2024). Furthermore, lysolecithins are potent surfactants that improve digesta homogeneity, decrease the size of emulsion droplets, and expedite the enzymatic breakdown of nutrients in the gastrointestinal tract. Consequently, lysolecithin is hypothesized to improve nutrient absorption through its involvement in the creation of micelles. Similarly, Drażbo et al. (2019) and Nemati et al. (2021) observed improved protein digestibility in turkeys when de-oiled lecithin was added to their diet. These authors suggested that lecithin may help regulate the equilibrium state of adsorption-desorption, influencing the presence of amphiphilic molecules like proteins, bile salts, and phospholipids at the interphase. However, Zhang et al. (2011) did not observe any effect on the nutrient retention of birds fed lysolecithin and other fat sources.

The non-effect of dietary lysolecithin on the haemato-biochemistry variables is the same as in the report of Cho et al. (2012) and Mohammadigheisar et al. (2018) who also observed no significant effect on the haematology parameters of broilers fed diets supplemented with emulsifiers and lysolecithin in various energy diets. Similarly, Gholami et al. (2024) reported no dietary effect on the blood biochmestry of broilers fed emulsifer supplemented diet in broiler chickens. These results are further corroborated by Singh et al. (2023), who found no dietary effect on the haematology and serum biochemistry of the broilers fed emulsifier at different energy levels. However, the report of Roy et al. (2010) showed that supplemetal emulsifier had variable effects on serum metabolites. The findings of the present study indicate that dietary lysolecithin does not induce physiological alteration in the animals.

Moreover, supplementation with lysolecithin at 300–400 g /100 kg increased the levels of SOD, GPx, and GSH, whilst reducing the MDA content in the breast meat, suggesting its protective role against oxidative stress. The findings of Zangeneh et al. (2020), who reported enhanced free radical scavenging activity and decreased lipid peroxidation in broilers fed dietary lysophospholipid under cold stress are consistent with this. Similarly, EL-Gendi et al. (2023) observed that dietary inclusion of de-oiled lecithin elevated the serum levels of SOD, GPx, and total antioxidant capacity (TAC), while reducing MDA concentrations in two different strains of broilers. Lysolecithin comprises gamma, alpha, and delta tocopherols, thus, it is believed that the main antioxidant function of lecithin is derived from the synergistic effect of amino-alcohol phospholipids and gamma and delta tocopherols (Judde et al. 2003; Wang and Wang 2008; EL-Gendi et al. 2023). The presence of polar headgroups, such as amino groups and choline, in phospholipids renders them appropriate for application as lipophilic antioxidants in food products, and can efficiently counteract oxidative stress and provide protection against the detrimental impacts of free radicals (Sun et al. 2018). Accordingly, Das and Vasudevan (2006) noted that lysolecithin exhibits antioxidant and neuroprotective properties, which contribute to its ability to reduce liver damage and enhance oxidative resistance. It also can enhance the resistance of oils and fats oxidation due to the presence of phospholipids, which are the main constituents of lecithin.

In conclusion, dietary lysolecithin supplementation induced positive effects on broiler chicken performance, nutrient digestibility, haemato-biochemistry, as well as antioxidant status and lipid peroxidation. Lysolecithin could therefore be supplemented up to 400 g /100 kg without compromising growth performance, nutrient digestibility, haemato-biochemistry, or antioxidant status in finisher broiler diets.

## Electronic supplementary material

Below is the link to the electronic supplementary material.

Supplementary Material 1

## Data Availability

The data will be made available on request.
