# Supplementary material for: Influence of dietary lysolecithin on growth performance, nutrient digestibility, haemato-biochemistry, and oxidative status of broiler birds
Source: Trop Anim Health Prod. 2024 Sep 23;56(8):271. doi: 10.1007/s11250-024-04107-7 (PMC11420263; doi:10.1007/s11250-024-04107-7)
Supplement: Supplementary file 1 — Supplementary Material 1 [file 11250_2024_4107_MOESM1_ESM.docx]

Food Security and Safety Focus Area,

Faculty of Natural and Agricultural Sciences,

North-West University,

Mahikeng Campus,

Mmabatho 2735, South Africa.

22^nd^ May, 2024

The Editor-in-Chief,

**Tropical Animal Health and Production**

**Re: Manuscript for consideration for publication**

Please find attached, our manuscript entitled “**Influence of dietary lysolecithin on growth performance, nutrient digestibility, haemato-biochemistry, and oxidative status of broiler birds** ” for consideration and publication in **Tropical Animal Health and Production**. This manuscript is not under consideration for publication elsewhere and its content and style have been approved by the authors. We hope that the information contained in this manuscript will be useful to all poultry farmers (industry) across the world. We also believe that all aspect of this manuscript will be of interest to the readers of **Tropical Animal Health and Production.**

Kindest regards

Mercy Chisara Ogwuegbu (Ph.D)
